# Dourine: a neglected disease of equids

**DOI:** 10.1007/s11250-017-1280-1

**Published:** 2017-04-24

**Authors:** Yonas Gizaw, Mulisa Megersa, Teka Fayera

**Affiliations:** College of Veterinary Medicine, Jigjiga University, P. O. Box: 1020, Jigjiga, Ethiopia

**Keywords:** Diagnosis, Dourine, Identification, Pathological lesions, *Trypanosoma equiperdum*

## Abstract

Dourine is a venereal transmitted trypanosomosis causing a major health problem threatening equines worldwide. The origin and identification of *Trypanosoma equiperdum* within the subgenus *Trypanozoon* is still a subject of debate. Unlike other trypanosomal infections, dourine is transmitted almost exclusively by coitus. Diagnosis of dourine has continued to be a challenge, due to limited knowledge about the parasite and host-parasite interaction following infection. The pathological lesions caused by the diseases are poorly described and are observed mainly in the reproductive organs, in the nervous system, and on the skin. Dourine has been neglected by research and current knowledge on the disease, and the parasite is very deficient despite its considerably high burden. This paper looks in to the challenges in identification of *T. equiperdum* and diagnosis techniques with the aim to update our current knowledge of the disease.

## Introduction

Dourine is a contagious disease of equids caused by the protozoan parasite *Trypanosoma equiperdum*. Once widespread, dourine has been eradicated from many countries but is still seen in horses in Asia, Africa, South America, Southern and Eastern Europe, Mexico, and Russia and was reported in June 2011 in Sicily and then just north of Naples, on the Italian mainland (Sidney et al. 2013).

*T. equiperdum* is morphologically identical to other insect vector-transmitted *Trypanosoma evansi* and *Trypanosoma brucei* which cause surra and nagana, respectively. In many regions of the world, these three parasite species occur together and current diagnostic tests are unable to differentiate between them (Brun et al. 1998). *T. equiperdum* differs from other trypanosomes in that it is primarily a tissue parasite that rarely invades the blood. The trypanosomes, which are present in the seminal fluid and mucous membranes of the genitalia of the infected donor animal, are transferred to the recipient during sexual intercourse. Parasites then may pass into the blood, where they are carried to other parts of the body. In typical cases, this metastatic invasion gives rise to characteristic cutaneous plaques (Stephen 1986).

In practice, diagnosis of dourine is based on clinical evidence supported by serology (Office International des Epizooties (OIE) 2008). Despite available serological tests are more sensitive, they fail to distinguish between an active infection and a cured one (Clausen et al. 1999). Detection of *T. equiperdum*, by standard parasitological techniques, is usually difficult even in dourine positive horses, due to low numbers of parasites in the blood or tissue fluids and chronic nature of the disease (Vulpiani et al. 2013). Additionally, diagnosis of the disease becomes more complicated in an area where the causative agents of surra or nagana occur and appears difficult to identify which parasite is causing dourine (Hagos et al. 2010b).

Dourine is a disease of great economic importance and well documented as a trade barrier for the movement of horses (Chin et al. 2013). Dourine poses a significant challenge to equine production, as transmission does not require insect vectors that are influenced by climatic factors, the disease can be found anywhere and even the disease is more important in areas where mechanically or tsetse-transmitted trypanosomes are endemic. Though dourine still occurs in many parts of the world, since its eradication from North America and Northern Europe, research on the disease has been neglected. The absence of published information on many aspects of dourine should prompt experts in the field to bridge the gap in current knowledge about the disease possibly through research or systematic review of existing literature. This paper presents a thorough review of the epidemiology, diagnosis, and pathology of dourine.

## Equine Trypanosomosis (dourine)

### Definition and synonyms

Dourine is a chronic or acute contagious disease of equids transmitted directly from animal to animal during coitus (Calistri et al. 2013). The venereal disease of equines or dourine has been also known under different other names (Arabic “*el Dourin*,” English “covering disease,” German “*Beschalseuche*,” French “*Mal de coit*,” Russian “*Slucnaja Boleznj*,” or “*Podsedal*”) (Hoare 1972).

### Etiology

*T. equiperdum* is the causative agent of dourine that belongs to the subgenus *Trypanozoon* (Hébert et al. 2017). This subgenus also includes the three subspecies of *T. brucei* (*Trypanosoma brucei brucei*, *Trypanosoma brucei gambiense*, and *Trypanosoma brucei rhodesiense*), and *T. evansi*. *T. b. brucei* causing nagana in domestic animals and *T. b. rhodesiense* and *T. b. gambiense* causing sleeping sickness in humans. Further, *T. evansi* causes surra predominantly in livestock but also in other mammals (Maudlin et al. 2004).

#### Origin and identification of the parasite

**Fig. 1:**
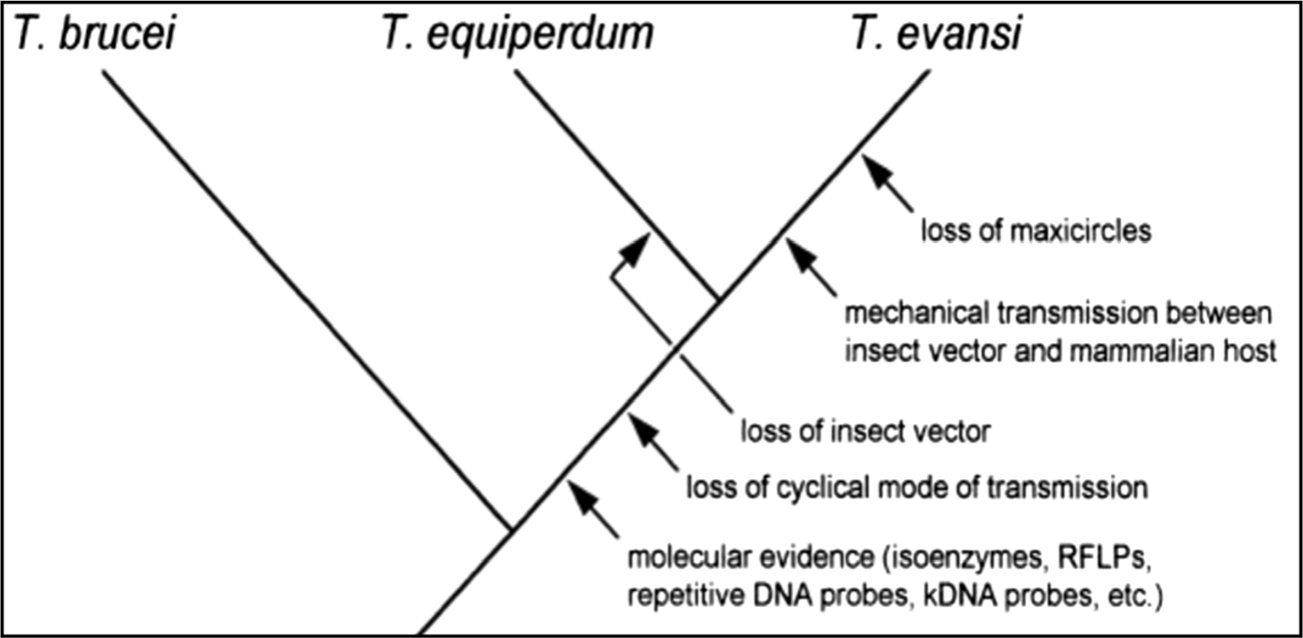
Phylogenetic relationship among three closely related trypanosomes which indicates the close relationship between *T. evansi* and *T. equiperdum* (Brun et al. 1998)

*T. equiperdum* is classified under the subgenus *Trypanozoon* along with *T. brucei* spp. and *T. evansi*; however, the species classification of *Trypanozoon* remains a controversial topic because it has been hypothesized that a very close evolutionary relationship exists among the trypanosome species of *Trypanozoon* (Suganuma et al. 2016). Based on biological and morphological characteristics, Hoare (1972) suggested that *T. evansi* evolved from *T. brucei* by adaptation to mechanical transmission from host to host through their insect vectors, while *T. equiperdum* was derived from *T. evansi* by adapting to the equine hosts. However, since *T. evansi* lacks kinetoplast DNA (kDNA) maxicircles (Lun et al. 1992), Hoare’s hypothesis that *T. equiperdum* arose from *T. evansi* is inappropriate. In fact, there is no evidence to indicate that the ability to reacquire kDNA occurred in trypanosomes or other kinetoplastids (Lun et al. 1992). On the other hand, based on the morphology and molecular data from a study on *T. brucei*, *T. evansi*, and *T. equiperdum*, other researchers suggested that *T. evansi* stocks distributed around the world were derived from a mutated clone population of *T. equiperdum* that lacked maxicircle kDNA (Lun and Desser 1995; Brun et al. 1998). To date, phylogenetic analyses show that *T. equiperdum* and *T. evansi* are not monophyletic and should therefore be considered as subspecies of *T. brucei*, a parasite causing sleeping sickness in humans and nagana in animals (Hébert et al. 2017). The possible phylogenetic relationship is illustrated in Fig. 1.

Unlike *T. brucei* whose kDNA contains hundreds of complex heterogeneous minicircle sequence, *T. equiperdum* and *T. evansi* share the same properties of minicircles which are largely homogeneous and totally different from that of *T. brucei* having only a single major minicircle class (Gibson 2007; Lai et al. 2008; Lun et al. 2010). This strongly supports the hypothesis that *T. evansi* is likely to directly arise from a mutated *T. equiperdum* which has a lack of maxicircles.

Based on the biological and molecular evidence, it is suggested that *T. equiperdum* evolved from an ancient strain of *T. brucei* which adapted to equine hosts, and that during the period of adaptation, some parts of the maxicircle kDNA and the heterogeneous minicircles were lost, causing the lack of development within an insect vector (Lai et al. 2008). This is supported by the deletion of maxicircle sequences observed in at least two stocks of *T. equiperdum* (Lun et al. 1992). Lun et al. (1992) showed that Chinese *T. equiperdum* maxicircles are only about half the size of those of *T. brucei*, being approximately 14.3 kb in size. Because of the lack of parts of the maxicircle kDNA, cyclic stages no longer occurred in this mutated trypanosome (Borst et al. 1987) and then this ancestral *T. brucei* isolate, later called *T. equiperdum*, was finally limited to the equine hosts.

Although great progress has been made in clarifying the genetic and evolutionary relationships, many interesting questions still need to be resolved with more evidence. In addition, it is not clearly understood how or how often maxicircle kDNA loss has happened in dyskinetoplastic trypanosomes although it is clear that this phenomenon is frequently observed in *T. evansi* and *T. equiperdum* both in vivo and in vitro. At the same time, the links between mechanical transmission, the loss of kDNA, and host specificity remain uncertain (Wei et al. 2011).

It is difficult to distinguish *T. equiperdum* microscopically from other members of the subgenus *Trypanozoon* (*T. evansi* and *T. brucei*). In particular, *T. equiperdum* and *T. evansi* cannot be differentiated on the basis of morphological criteria (Claes et al. 2005). Like *T. evansi*, *T. equiperdum* is usually monomorphic. However, it sometimes exhibits pleomorphism like *T. evansi* during subpassages in rodents (Wei et al. 2011). At the fine structural level, there are relatively more coated vesicles in the flagellar pocket of *T. equiperdum*, compared with that of *T. evansi*. It becomes somewhat difficult to differentiate these two species with respect to the ultrastructural properties (Brun et al. 1998).

Neither parasitological nor serological tests are sensitive and specific enough, thus leading to various kinds of genetic and molecular methods which have been continually updated in order to enhance greater precision in diagnosis of *Trypanozoon* species and differentiation of these pathogens (Wei et al. 2011). Accordingly, restriction fragment length polymorphisms (RFLPs) (Lun et al. 2004), genome fingerprinting (Waitumbi and Murphy, 1993), and repetitive DNA probes were used (Zhang and Baltz 1994). A series of techniques based on PCR have also been used, for example, mini satellite DNA analysis (Macleod et al. 2001), amplified fragment length polymorphism (AFLP) (Agbo et al. 2002), multiplex-endonuclease genotyping (MEGA) (Claes et al. 2003), mobile genetic elements (MGE)-PCR, simple sequence repeat (SSR)-PCR (Li et al. 2005), and random amplification of polymorphic DNA (RAPD) (Lun et al. 2004). PCR test based on the RoTat1.2 variable surface glycoprotein (VSG) cDNA sequence was performed by Claes et al. (2004). Moreover, two kinds of techniques have been developed for detection and identification of African trypanosomes, i.e., fluorescence in situ hybridization with peptide nucleic acid probes (Radwanska et al. 2002) and the loop-mediated isothermal amplification (LAMP) reaction (Thekisoe et al. 2007; Njiru et al. 2008). However, despite the development of these, genetic and molecular techniques by different scholars to clear species-specific identification within the subgenus trypanosome remains difficult. So far, the discovery of a simple and reliable way to entirely distinguish all *Trypanozoon* species remains a big challenge.

### Epidemiology

#### Host range and geographical distribution

*T. equiperdum* has been reported to infect horses, donkeys, and mules. There is no known natural reservoir of the parasite other than infected equids (Brun et al. 1998). Infection is not always transmitted by an infected animal during copulation (OIE 2013). Horses usually die from infection without treatment, whereas donkeys and mules are more resistant than horses and may remain unapparent carriers. Zebras have been tested positive by serology, but there is no conclusive evidence of infection (Brun et al. 1998). Since *T. equiperdum* is a tissue parasite found in equines in nature, its establishment in the blood of laboratory animals is extremely difficult. However, once a strain becomes adapted to rodents, the parasites can be maintained by serial passages, in the same manner as *T. evansi*. It is noted that murine-adapted clones of *T. equiperdum* can cause acute infection like *T. evansi* when passaged through mice, rats, rabbits, horses, and dogs. Domestic animals such as sheeps and goats infected with murine-adapted strain of *T. equiperdum* produce the clinical manifestations of dourine (Wang 1988).

**Fig. 2:**
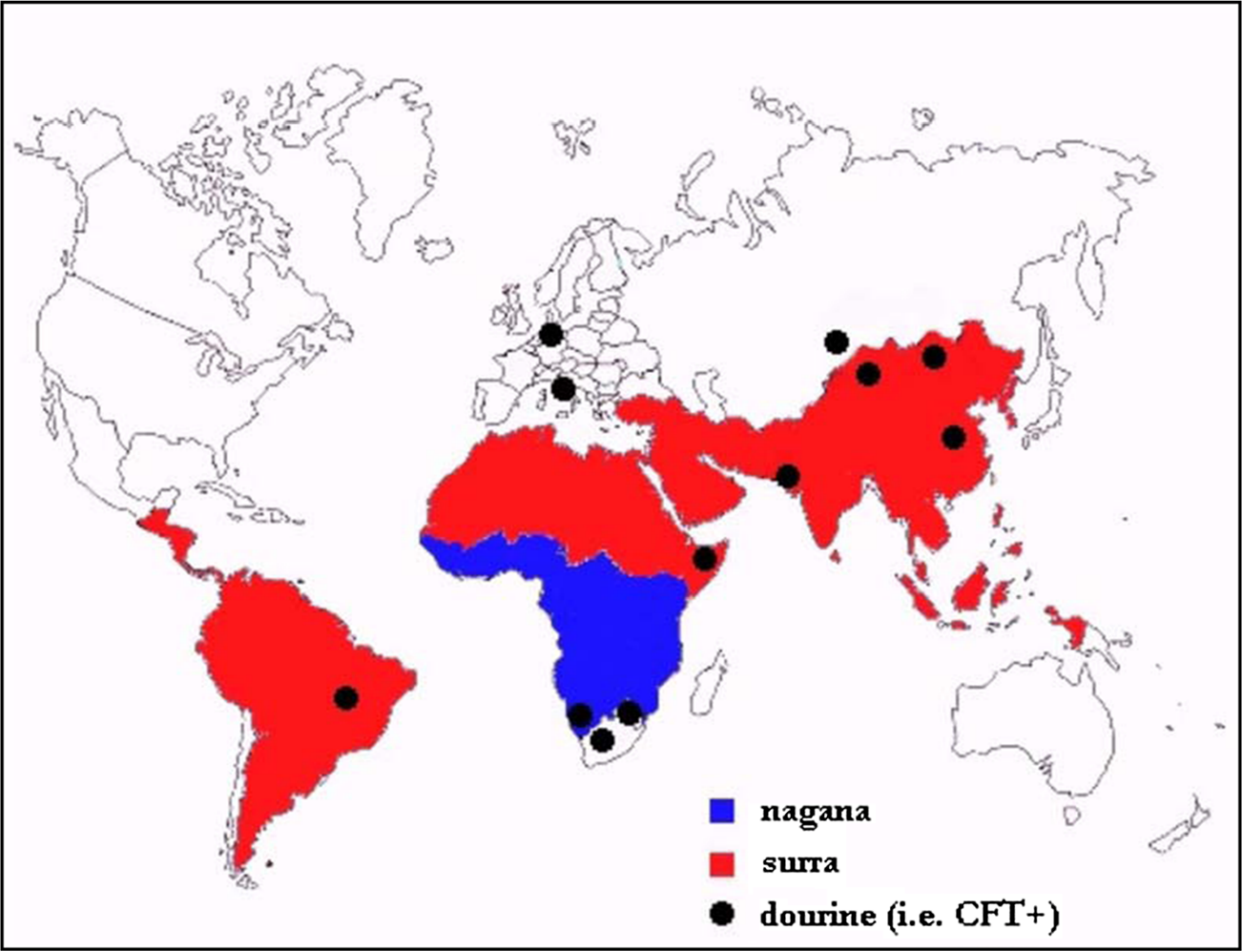
Distribution of dourine in different parts of the world (Yonas 2015)

Dourine has a worldwide distribution but few cases have been reported during the last three decades owing to the wide use of artificial fertilization technology (OIE 2013). It was once widespread during the times when the horse was militarily, economically, and agriculturally important. It was of great concern in the USA and Canada at the beginning of the twentieth century. Nowadays, Western Europe, Australia, and the USA are considered to be free from dourine (Claes et al. 2003). The infection is endemic in many areas of Asia, Africa, Russia, Middle East, and Eastern Europe (OIE 2008). The latest official reports of dourine (i.e., Complement Fixation Test (CFT) positive cases) were in China, Kazakhstan, Pakistan, Ethiopia, Botswana, Namibia, South Africa, Brazil, Italy, and Germany (Fig. 2). However, due to possible cross reactions in the CFT, it is difficult to conclude that seropositive animals are real *T. equiperdum* cases (Zablotskij et al. 2003)*.* The prevalence of the disease in some countries is summarized in Table 1.

**Table 1:** Prevalence of dourine in horses in some countries based on different tests

Countries	Test employed	Prevalence (%)	Source
Botswana	CFT	9.0	(Masupu and Majok 1998)
Ethiopia	ELISA	19.26	(Hagos et al. 2010a)
Ethiopia	Woo test, ROTat 1.2, and 18S PCR	4.6, 36.7, and 47.6	(Fikru et al. 2010)
Kazakhstan	CFT	16.4	(Claes et al. 2005)
Mongolia	CFT and ELISA	7.6 and 6.7	(Clausen et al. 2003)
Namibia	CFT	8.33	(Kumba et al. 2002)
Italy	CFT	0.54	(Calistri et al. 2013)

#### Transmission

Unlike other trypanosomal infections, dourine is transmitted almost exclusively during coitus. Dourine is the only trypanosomosis that is not transmitted by an invertebrate vector. *T. equiperdum* differs from other trypanosomes in that it is primarily a tissue parasite that is rarely detected in the blood (OIE 2013).

The trypanosomes, which are present in the seminal fluid and mucous membranes of the genitalia of the infected donor animal, are transferred to the recipient during sexual intercourse. Trypanosomes are rarely observed in the bloodstream of the host because they are normally localized in the capillaries of the mucous membranes of the urogenital tract. However, a few trypanosomes occasionally appear in the peripheral blood of animals with chronic infection. This could provide the opportunity for bloodsucking insects to mechanically transmit this parasite, although this is considered to be very rare (Wang 1988).

The infection is more commonly transmitted from stallion to mare, facilitated by the presence of the parasite in the seminal fluid and mucous exudates of the penis and its sheath. From the infected mare, the infection is transmitted to a stallion due to the presence of the parasite in the vaginal mucus (OIE 2013). A study conducted using clinical findings and laboratory and epidemiological analyses of the outbreaks in Italy, based on features such as prevalence, age, reproductive activity, and relationship between the affected animals, indicated that the infection is transmitted directly from animal to animal during coitus (Calistri et al. 2013). As the disease progresses, trypanosomes periodically disappear from the urethra or vagina; during these periods, the animals are non-infective. Non-infective periods may last for weeks or months and are more likely to occur in the later stages of the disease. Thus, transmission is most likely in the early disease process (Wang 1988). An interesting finding in the literature was a positive PCR test result from a prepuce swab taken from a dourine-free stallion immediately after mounting an infected mare. The horse remained negative at all subsequent tests, supporting the theory that the parasite is present in the genital tissues but that sexual transmission is not constant (Vulpiani et al. 2013).

*T. equiperdum* can pass through intact mucous membranes and it is possible for foals to acquire infection by contamination of nasal or conjunctival membranes with the vaginal discharge. These infected foals can spread the organism when they mature. Other means of transmission may also be possible, but there is no evidence that arthropod vectors play any role in transmission. Intravenous or intraperitoneal experimental infections suggest that mechanical transmission by bloodsucking flies cannot be excluded. Foals born to mares infected with *T. equiperdum* may be infected in utero or may become infected during parturition. Transmission to foals by ingestion of infected colostrum or milk is considered rare (William and Steven 2007). The presence of trypanosomes in the mammary secretions may support that the infection can occasionally pass to foals during suckling (Pascucci et al. 2013). Foals that ingest colostrum from infected mares will become seropositive due to passive transfer of antibodies; these foals are usually seronegative by from 4 to 7 months of age (William and Steven 2007).

### Clinical signs

The incubation period between exposure and initial clinical signs is highly variable; it may be as short as 1–2 weeks or as long as several years (William and Steven 2007). Clinical signs of dourine are highly variable in manifestation and severity. The disease is characterized mainly by swelling of the genitalia, cutaneous plaques, and neurological signs but severity varies with the virulence of the strain, the nutritional status of the horse, and stress factors. Clinical signs often develop over weeks or months, frequently waxing and waning with relapses, probably precipitated by stress. This can occur several times before the animal either dies or experiences an apparent recovery. The mortality rate is believed to be in excess of 50% (Sidney et al. 2013).

A number of authors have broken the course down into three stages: stage 1 (genital lesions), stage 2 (cutaneous signs), and stage 3 (nervous signs). Stage 1 involves genital edema and swelling, manifesting 1–2 weeks after infection. In stage 2, typical cutaneous plaques (“silver dollar” plaques) appear, with thickening of the skin, considered pathognomonic by some authors. Stage 3 is characterized by progressive anemia, neurological disorders, and paresis of the hindquarters, often ending in death (Claes et al. 2005).

A pathognomonic sign is the edematous plaque consisting of an elevated lesion in the skin, up to 5–8 cm in diameter and 1 cm thick. The plaques usually appear over the ribs, although they may occur anywhere on the body, and usually persist for between 3 and 7 days. They are not a constant feature. Pyrexia is intermittent; nervous signs include incoordination, mainly of the hind limbs, lips, nostrils, ears, and throat. Depigmentation of the genital area, perineum, and udder may occur. In the stallion, the first clinical sign is a variable swelling involving the glans penis and prepuce. The edema extends posteriorly to the scrotum, inguinal lymph nodes, and perineum, with an anterior extension along the inferior abdomen. In stallions of heavy breeds, the edema may extend over the whole floor of the abdomen (OIE 2013).

An observation made by Vulpiani et al. (2013) indicates that infected stallions revealed mild signs than the infected mares. Six months after infection, the stallions were almost asymptomatic. However, the differences with respect to sex cannot be statistically examined because of the low number of considered cases in the study. An observation made by Watson (1920) indicates that apart from the fact of increasing virulence resulting from continued passages accords with general experience that the disease is usually more progressive in the stallion than in the mare.

### Pathological lesions of dourine

The disease is characterized by edematous lesions of the genitalia, involvement of the nervous system, and progressive emaciation, and it is ultimately fatal in most cases. Typical cutaneous lesions, from which the disease derives its name “dourine,” have been described as circular elevated plaques of thickened skin ranging in size from 1 to 10 cm in diameter, resembling money or “douros” (Claes et al. 2005). The constant antigenic variations of the parasite result in the release of a large amount of biological active products and the formation of immune complexes, which are certainly major factors in triggering a variety of clinical and pathological changes (Zwart 1989).

#### Gross pathological lesion

Dourine is characterized by cachexia, anemia, muscular hypotrophy, ataxia, and lack of coordination of the hindquarters, ptosis of the lower lip, genital lesions, skin edematous plaques, and peripheral edema (Pascucci et al. 2013). The presence of nervous signs without sensory alterations seems to confirm the tropism of *T. equiperdum* for the peripheral rather than the central nervous system, in contrast with other trypanosomes (Berlin et al. 2009).

At postmortem examination, gelatinous exudates are present under the skin. In the stallion, the scrotum, sheath, and testicular tunica are thickened and infiltrated. In some cases, the testes are embedded in a tough mass of sclerotic tissue and may be unrecognizable. In the mare, the vulva, vaginal mucosa, uterus, bladder, and mammary glands may be thickened with gelatinous infiltration. The lymph nodes, particularly in the abdominal cavity, are hypertrophied, softened, and, in some cases, hemorrhagic. The spinal cord of animals with paraplegia is often soft, pulpy, and discolored, particularly in the lumbar and sacral regions (OIE 2013).

The presence of dourine infection in the stallions did not appear to interfere with libido or the ability to achieve erection even where there is pronounced edema of the scrotum and sheath. Similarly, the presence of infection did not appear to adversely affect the fertility of either stallions or mares. This study also reported on five occasions clean mares conceived to services by infected stallions and on three occasions infected mares conceived to services by clean stallions. Two foals born to infected mares were normal and were reared to maturity (Barrowman 1976).

#### Microscopic lesions

On histological examination of tissue samples, the disease is characterized by hemosiderin deposition in the spleen, the iliac, supramammary, and popliteal lymph nodes showed non-specific reactivity with hyperplasia of the plasma cells, a sign of increased hemolymphatic activity. The edematous plaque showed a characteristic picture of pustular dermatitis, particularly severe around the lesion, with severe inflammation and vacuolar degeneration extending to the deepest layers of the skin, with involvement of the cutaneous adnexa and perivascular plasma cell inflammation. There was exudates of cell detritus in the same area, mainly eosinophils and the bodies of free parasitic protozoa, in a picture described as “trypanosomal sand” (Pascucci et al. 2013; Scacchia et al. 2013).

In the nervous system of infected horses, neurodegenerative lesions and inflammatory vasculitis of the central nervous system with edematous infiltration in the facial and lingual nerves were reported. In the udders, there are histological lesions attributable to severe interstitial inflammation accompanied by strong supramammary lymph node reactivity and the presence of Russell’s bodies. Liver showed multifocal areas of hepatitis while the kidneys are affected by plasma cell inflammation of the renal pelvis. Periglandular inflammation in the vulva, vagina, uterus, and clitoris was also observed in the infected horse. The constant finding of iliac and supramammary lymph node positivity and lymphatic activity on both macroscopic observation and histological examination seem to confirm that the parasite spreads mainly through the lymphatic system (Pascucci et al. 2013).

**Fig. 3:**
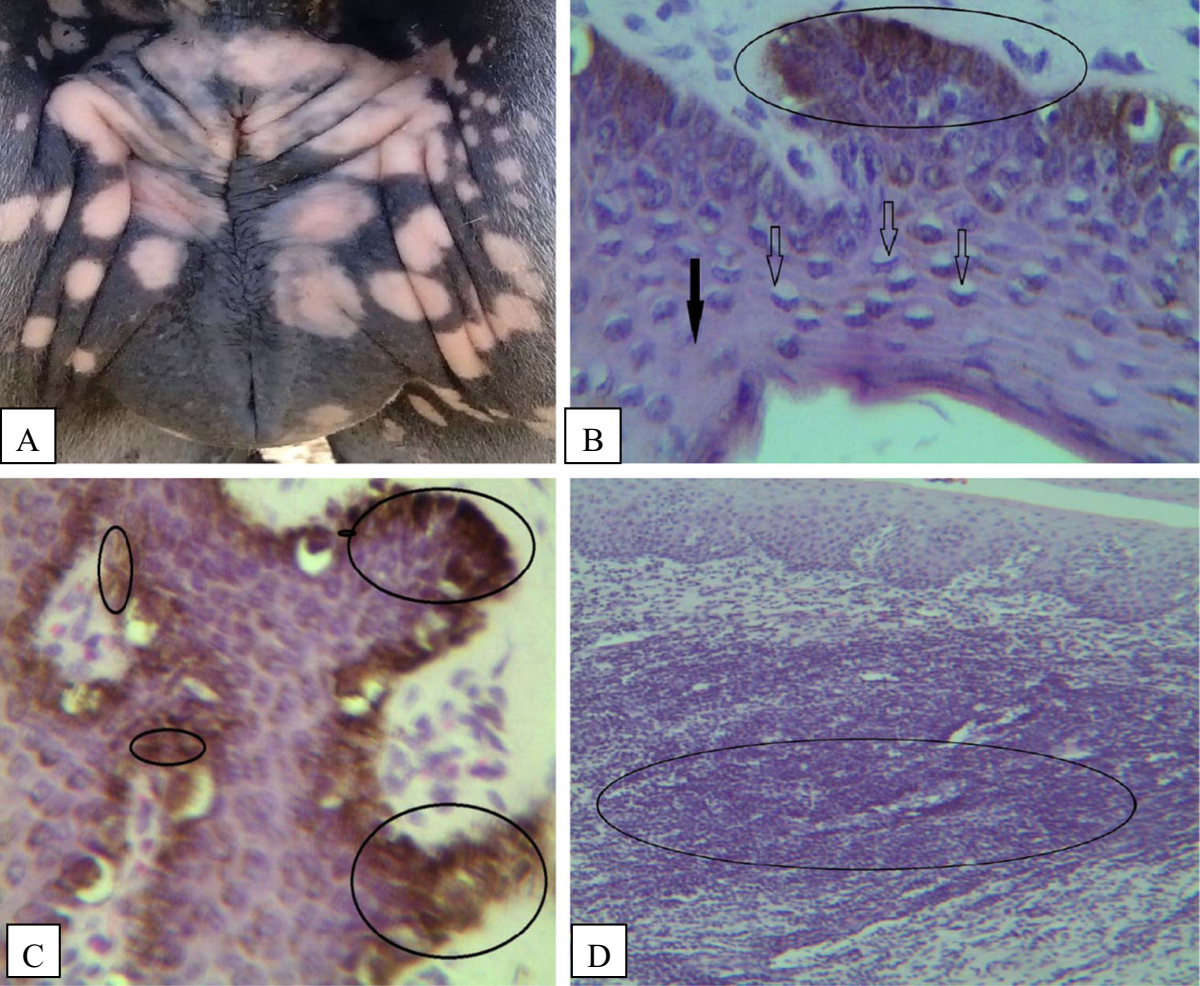
**a** Depigmentation of the vulval lip (*gross*). **b** Vacuolar degeneration of the cells (*lighter arrows*) and necrotized cell (*darker arrow*) in the stratum spinosum, degeneration and necrosis of the basal cells with melanin pigment were evident (*circled areas*). **c** Excess free melanin in the stratum spinosum (*small circles*) and within the basal layer (*large circles*). **d** Severe dermatitis with infiltration of lymphocytes and plasma cells in the epidermis and dermis (*circled areas*) (Yonas 2015)

Although, depigmentation around the perineum is often described as characteristic of clinical cases of dourine (Claes et al. 2003; Hagos et al. 2010a; Vulpiani et al. 2013), no microscopic description of such lesions was cited in previous literatures. Severe dermatitis with hydropic degeneration and necrosis of the keratinocytes of stratum spinosum and necrosis of basal cells including the melanocytes with excess free melanin pigment within the epidermis were reported recently. The probable cause of depigmentation around the vulval skin of infected mares could be due to severe necrosis of melanocytes, as the depigmented areas were microscopically characterized by severe necrosis of cells, excess free melanin, and formation of cystic structures in the epidermis (Yonas 2015) (Fig. 3).

### Diagnosis

Diagnosis of dourine is a challenge, due to limited knowledge about the parasite and host-parasite interaction following infection. In practice, diagnosis is based on clinical evidence supported by serology (Alemu et al. 1997; Hagos et al. 2010a). Clinical signs of dourine can provide a strong indication of the presence of the disease, but confirmatory diagnosis is needed (Claes et al. 2005). The incubation period may vary from a few weeks to several years, and some of the clinical signs, which include genital edema, weight loss, skin lesions known as silver dollar plaques, and neurological signs, may be absent in the early stages or during latent infections (Luckins et al. 2004; Claes et al. 2005). Diagnosis of dourine, therefore, requires confirmation by parasitological, serological, and molecular techniques.

#### Parasitological diagnosis

##### Wet and thick blood films

In this test, 5–10 μl of blood is placed on a slide and examined microscopically at ×400 magnification under a cover slip. Trypanosomes are observed moving between the erythrocytes in infected animals. It has very low sensitivity, with a detection limit as high as 10,000 trypanosomes/ml, but it is still in use because of its low cost and simplicity. Giemsa or Field-stained thin blood films have a similarly low sensitivity. It is time consuming (10–20 min per slide) and requires expertise to recognize the parasite (Murray et al. 1979).

##### Microhematocrit centrifugation technique

Microhematocrit centrifugation technique (mHCT), a blood concentration technique (also called the capillary tube centrifugation technique or the Woo test), is the most frequently applied concentration technique with better sensitivity than direct microscopic examination. In this test, capillary tubes containing anticoagulants are filled three-quarters full with blood. The dry end is sealed with plasticine. By high-speed blood centrifugation in a hematocrit centrifuge for 6–8 min, trypanosomes are concentrated between the red blood cells and the plasma, together with the white blood cells. The capillary tubes mounted in a special viewing holder can be directly examined at low magnification (×10 or ×40) for motile parasites. The estimated detection threshold of mHCT is 500 trypanosomes/ml of blood sample (Reid et al. 2001).

##### Mini anion-exchange centrifugation technique

The mini anion-exchange centrifugation technique (mAECT) consists of separating the trypanosomes which are less negatively charged than blood cellular components from venous blood via anion-exchange chromatography and finally concentrating them at the bottom of a plastic collector tube by low-speed centrifugation. The tip of the glass tube is then examined in a special holder under the microscope for the presence of trypanosomes. The large blood volume of up to 300 μl enables the detection of less than 100 trypanosomes/ml, resulting in high sensitivity. However, the manipulations are quite tedious and time consuming (Reid et al. 2001; Buscher et al. 2009).

##### Animal inoculation

Repeated attempts have been made by different workers (Alemu et al. 1997; Clausen et al. 1999, 2003) to demonstrate and isolate *T. equiperdum* in laboratory mice but all were unsuccessful. However, once a strain becomes adapted to rodents, the parasites can be maintained by serial passages, in the same manner as *T. evansi* (Luckins 1994). Under laboratory conditions, dogs can be infected with *T. equiperdum* as reported by Rouget (1986). In experimental infections carried out in the Institute for Tropical Medicine to raise antisera against VSGs, rabbits infected with the available laboratory strains developed clinical signs that could not be distinguished from those developed by rabbits infected with *T. evansi* (Verloo et al. 2001). Owing to the marked predilection of *T. equiperdum* for the testicles of rabbits, some authors recommended intratesticular inoculation of these animals for the diagnosis of dourine in equines. Ruminants were refractory to infection with *T. equiperdum* (Hoare 1972).

#### Serological techniques

It is extremely difficult to detect the parasite in the body fluids of infected horses (Claes et al. 2005); therefore, diagnosis of *T. equiperdum* by standard parasitological techniques is difficult, owing to the low numbers of parasites in the blood or tissue fluids. Consequently, the demonstration of trypanosomal antibodies in the serum has become the most important parameter determining the disease status of individual animals (Bishop et al. 1995). *Trypanozoon* group-specific trypanosomal antigen could be of use in an antibody assay for the diagnosis of *T. equiperdum* infections. However, based on anecdotal evidence, it appears that *T. equiperdum-*infected laboratory animals and horses suspected of dourine also positively react in the Card Agglutination test trypanosomiasis (CATT)/*T. evansi* and Enzyme- linked Immunosorbent Assay (ELISA)/*T. evansi* prepared with fixed whole trypanosomes of the RoTat 1.2 VAT (Claes et al. 2003).

CATT/*T. evansi* test is fast, uses a standardized antigen, and can be performed in situ, i.e., without the need of a fully equipped laboratory. Recently, it has been proven that most so-called *T. equiperdum* strains also express isoVATs of *T. evansi* RoTat 1.2. Therefore, the CATT/*T. evansi* may prove to be a good test for equine trypanosomosis, regardless whether the causative agent is *T. evansi* (surra) or *T. equiperdum* (dourine) (Claes et al. 2003).

The complement fixation test is the most commonly used OIE-prescribed serodiagnostic test developed for *T. equiperdum* and successfully used as part of a program to eliminate *T. equiperdum* from North America. It is still used for international trade in monitoring horses for export/import. Despite the usefulness and universal acceptance of the CFT for diagnosing dourine, some discrepancies have been recorded. The disadvantages of the CFT are that it requires careful continuous titration of numerous labile agents and that it does not function with sera having anticomplementary activity. CFT is not species specific, but only specific for the subgenus *Trypanozoon*. The drawback of the test is lower specificity where it cannot differentiate *T. equiperdum* from other similar trypanosomes. Hence, the diagnostic significance of CFT is therefore doubtful in countries where both *T. equiperdum* and *T. evansi* infections occur in equines (Luckins 1994). Although the CFT has been in use for many years for diagnosis of dourine, it is considered to be less sensitive than ELISA and IFAT for the detection of the serum antibodies against *T. equiperdum* (Wassal et al. 1991; Bishop et al. 1995).

Indirect fluorescent antibody test is frequently used for the diagnosis of dourine, as a confirmatory test for CFT results, since immunofluorescence provides a reliable and sensitive technique. But its interpretation is both subjective and labor intensive, and it is therefore more suited to the testing of small numbers of sera (Williamson et al. 1988).

The use of ELISA for routine diagnosis of dourine would provide a significant advantage over current serological tests if a defined antigen was used, since it would permit test standardization and more readily allow comparison of tests among laboratories. It additionally, lends itself to a considerable degree of automation, which makes it suitable for a large number of samples (Wassal et al. 1991). Different workers have stated that the ELISA has a satisfactory concordance ratio with CFT and can be used to supplement CFT (Williamson et al. 1988; Alemu et al. 1997). There are also several other alternative serological tests that are used, such as the agar gel immunodiffusion test, the arrayed immunodiffusion method (Hagebock et al. 1993), and the competitive immunoassay (cELISA). The cELISA method has several advantages over the CFT: it can be performed in less time than the corresponding CFT procedure, it is reproducible, results are objectively measured and calculated, and the method is amenable to automation (Katz et al. 1999). While serological tests can be the method of choice for mass screening of populations, their main limitation will remain as the failure to demonstrate the parasite. Unfortunately, parasitological techniques are known to lack sensitivity, especially for the detection of *T. equiperdum*, which is considered to be a tissue parasite rather than a blood parasite (Brun et al. 1998).

#### Molecular techniques

Although no *T. equiperdum-*specific polymerase chain reaction (PCR) method is available, subgenus *Trypanozoon*-specific PCR can be used for detection of *T. equiperdum* DNA. Recently, a highly sensitive real-time PCR for *Trypanozoon* subgenus was applied on tissues and fluid samples from a naturally dourine-infected horse, enabling the detection of low numbers of the parasites (Scacchia et al. 2011; Pascucci et al. 2013). PCR and other related DNA amplification methods have been used to examine exudates or tissue samples, taking into account their failure on blood samples after the initial phase of the infection (Calistri et al. 2013).

Direct diagnosis based on molecular techniques can be highly sensitive for parasite detection in body fluids such as the blood (Becker et al. 2004). However, this approach is difficult to apply for mass screening and negative results do not exclude the possibility of infection. In fact, *T. equiperdum* multiplies predominantly in extracellular tissue spaces and is seldom found in peripheral blood (Theis and Bolton 1980). Diagnosis of *T. equiperdum* infection is thus still strongly based on serological evidence.

### Treatment

Pharmaceutical therapy is not recommended because animals may improve clinically but remain carriers of the parasite (OIE 2013). There are no officially approved drugs to treat horses suffering from dourine although some older publications mentioned experimental treatment of horses with suramin and neoarsphenamine (Ciuca 1933) or quinapyramine sulfate (Vaysse and Zottner 1950). Evidence from in vitro drug sensitivity determination of *T. equiperdum* indicates that suramin, diminazene, quinapyramine, and cymelarsan are effective (Zhang et al. 1992; Brun and Lun 1994). These were also supported by other researchers who found cymelarsan® quite effective in curing horses at both 0.25 and 0.5 mg/kg in acute as well as chronic form of dourine (Hagos et al. 2010b). Brun and Lun (1994) reported drug sensitivity of *T.equiperdum* isolates in vitro and found the isolate was highly sensitive to melarsorol, isometamidium, and suramin; with regard to diminazene, *T.equiperdum* was not sensitive as the most sensitive *T. evansi* strains.

### Prevention and control

There is no vaccine available for dourine. As dourine is primarily a venereal disease, prevention of natural mating or AI with infected horses (stallions or mares) or infected stallion semen is the most important means of control. Prevention of dourine is therefore based on the establishment of freedom from infection, and this is done by testing blood for the presence of antibodies against *T. equiperdum*, which is more reliable than testing for the presence of the protozoan parasite itself. Any introductions of horses from endemic areas or areas of incursion should be isolated and the blood tested for antibodies by complement fixation test (Sidney et al. 2013).

Control of the disease depends on compulsory notification, slaughter of infected animals, and movement control enforced by legislation in most countries (OIE 2013). Dourine should be eradicated from an incursion into a non-endemic area by identification of the source, thorough tracing and testing of all in contact, and euthanasia of infected and seropositive horses (Sidney et al. 2013). Currently, an eradication strategy is imposed by the World Organization for Animal Health (OIE) with slaughtering of seropositive horses while treatment is prohibited (Zablotskij et al. 2003). However, it is not economically feasible to apply a strict test and slaughter policy to control dourine in developing countries. Based on the result of the in vivo drug sensitivity study, a revised strategy of the appropriate drug treatment in dourine endemic areas instead of eradication could be recommended to the OIE (Hagos et al. 2010b).

It is important to note that castrating adult stallions does not always change the copulatory ability of such animals and it should be performed with caution when attempting an eradication program. To prevent the introduction of dourine, serum samples should be taken following a period of isolation (quarantine) to ensure that the animals are not in the incubation period (Zablotskij et al. 2003).

The difficulty in the diagnosis of *T. equiperdum* has led to difficulties in obtaining reliable data on the prevalence and distribution of the disease and for the implementation of monitoring, treatment, and control program. Moreover, shortages of trypanocidal drugs and the absence of vaccines against trypanosomosis have hampered the control and prevention of the disease in endemic areas (Clausen et al. 2003).

## Conclusion

Owing to the difficulties and challenges related to the diagnosis of *T. equiperdum*, it was not possible to achieve reliable data on many aspects of the disease and above all for the implementation and monitoring of the disease control program. Similarly, the less attention given to study the disease resulted in a remarkable deficiency in our current knowledge of the disease. Hence, dourine imposes further detail study in developing very sensitive and specific diagnostic tools, host parasite interaction (pathology), and chemotherapy which will have tremendous aid in effective control of the disease.
